# Diseases of the Stomach, Liver, and Intestines

**Published:** 1903-08-15

**Authors:** 


### Diseases of the Stomach, Liver, and Intestines.

**Digestive Organs.**—Russell[1] has drawn attention to cases of "acid dyspepsia," frequently resulting in gastric catarrh and dilatation, which are caused by an excess of HCl. and not by the organic acids produced in fermentation (hyperchlorhydria). The symptoms are pains of varying character referred to the epigastrium, the back, either shoulder blade, the position of the normal pylorus or over the liver behind; these occur an hour or more after food and are sometimes temporarily relieved by the next meal or by a little acid vomiting. The patients may also suffer from heartburn, exhaustion, and mental depression. An accurate history can only be elicited by careful questions and often gives the clue to the diagnosis. The condition is due to the retention in the stomach of unaltered fat and starch after the proteids have passed into the duodenum. This causes continued secretion of HCl, resulting in pyloric spasm (paroxysmal pyrosis). There is probably in some cases (especially in the gouty and rheumatic) a true "acid dyscrasia," but the condition is also caused by errors in diet alone. It is essentially *not* a neurosis. The treatment is to correct the frequently associated constipation, to exhibit alkalis either before food or when pain occurs, to limit or forbid carbohydrates and fats, and to exclude the less easily digested proteids. Removal of the acid contents of the stomach is useful temporarily, and the food taken should not be too bulky. The *Lancet*[2] summarises the literature of gastric ulcer and its causes, and reviews Dr. R. Dalla Vedova's recent experiments. By resection of the solar plexus and vagi he was able to produce hæmorrhages, ulceration and necrosis, chiefly in the antrum pylori, in about half the animals operated upon. Peritonitis and mechanical injuries could be excluded in all cases. He supposes the lesions to be due to irritation by the acid of an already debilitated mucous membrane. H. W. Bayly[3] reports three cases of perforating gastric ulcer. In the first perforation occurred without pain or any immediate symptoms; post-mortem there was a hæmorrhage filling the lesser peritoneal sac. In the second case acute septic peritonitis occurred without pyrexia, vomiting, or abdominal tenderness, the perforation caused no collapse, and there was no dyspeptic history. In the third case perforation actually produced pyrexia and the abdominal facies and general condition of the patient were the most important signs. Melæna also occurred, the bleeding point being below a cicatricial contraction in the stomach. Bendersky[4] has described the descent of the lower margin of the stomach when a patient changes from the horizontal to the upright position as characteristic of gastroptosis, and absent in either normal or dilated stomachs. The variation is from 2–4 cm. Some interesting instances of human ruminants are quoted in the *Lancet*.[5] These occurred in a father and two sons; the former had an hour-glass stomach, but this is unusual. Mimicry could be excluded. About 100 cases have been recorded since Fabricius in 1618 first described the condition. Howship Dickinson[6] considers three factors of importance in altering the condition of the tongue in disease:—(1) Deficiency of saliva, which somehow prevents the lingual epithelium drawing its proper nourishment from the blood; (2) pyrexia, which up to about 104° increases cell growth and above that point inhibits it; (3) want of friction and wash owing to failure of mastication and deficiency of saliva. Inflammation and congestion also alter the mucous membrane to some extent. The changes produced by these agencies may be either increase or diminution of the lingual mucous membrane. Under the former he classes (1) the spotted tongue (unimportant clinically); (2) the coated tongue which occurs in slighter illnesses. Under this heading are included the "strawberry" tongue (part of the scarlatinal rash) and the "plastered" tongue which occurs in recent acute fevers. (3) The furred or shaggy tongue found in chronic cases especially when solids have been prohibited, and due mostly to disuse; (4) the encrusted, brown, dry tongue of grave omen, and generally found in long illnesses accompanied by pyrexia and exhaustion. Under the head of "diminution" comes (5) the red smooth dry tongue found in wasting and exhausting conditions such as prolonged suppuration. The tongue, Dr. Dickinson says, is an index of disease, but not of its locality; it is valuable both in prognosis and treatment.

Golla[7] has estimated the acetone bodies excreted by 11 patients under treatment for gastric ulcer, and therefore partially starved. There was a marked relation between the loss of body fat and the increase in acetonuria; the latter symptom was more marked than in Magnus Levy's cases and equalled occasionally the degree found in diabetic coma. The alkalinity of the blood was also diminished. He ascribes acetonuria to a disturbance in fat metabolism during starvation, and thinks diabetic coma probably caused by $\beta$-amidobutyric acid as suggested by Sternberg.

**Liver.**—Robinson,[8] divides cirrhosis into atrophic, hypertrophic and syphilitic, besides minor forms. The hypertrophic is differentiated from the atrophic by its morbid histology (a fine monolobular cirrhosis), causation (infection rather than alcohol) and symptoms (jaundice, fever, delirium, etc., but no ascites). It constitutes a separate disease (Hanot's disease), but is rare in typical form. Syphilitic cirrhosis may resemble either hypertrophic or atrophic cirrhosis, but is distinguishable anatomically and by the history. The diagnosis may be aided by a blood count and the results of treatment. The condition is rare in congenital cases. Malarial cirrhosis is also rare and may be partly alcoholic in some cases. No symptom is invariable in any form of cirrhosis. Atrophic cirrhosis, especially with ascites, is the most serious; in syphilitic and hypertrophic cases the prognosis is better if treatment can be instituted. The indications are abstinence from alcohol, careful dieting, and intestinal antiseptics to prevent auto-intoxication. Potassium iodide should be given in syphilis and where perihepatitis and other abdominal complications exist. Digitalis, salines and diuretics according to the symptoms and calomel in hypertrophic cases are useful. Paracentesis should be repeated as often as necessary. The surgical treatment by exciting adhesions with the development of collateral circulation is scientific; 55 cases have already been recorded. Ferris and Clayton Green[9] give a detailed account of a case of acute yellow atrophy with autopsy. The morbid histology is detailed. The view that the disease is an acute toxæmia is adopted though no micro-organisms were found. The condition is not local but general—a hæmolysis whereby a toxin capable of damaging various viscera is formed. The pigment found is not formed in the liver. Azzurrini[10] describes characteristic changes in the spleen observed in 20 cases of ordinary cirrhosis; they are localised in the pulp where the veins and lacunæ are dilated without increase of reticulum except in old cases. Banti's disease is contrasted.

**Dysentery.**—Rogers[11] states that the lesions in amœbic dysentery can be easily distinguished from the ordinary bacillary form and illustrates the appearances by a coloured plate. Preserved specimens lose their characteristic appearance. He considers (1) That amœbic dysentery is often latent, is chronic, is the cause of large "tropical" abscess, and is only fatal through complications. (2) These are large hepatic abscess, acute or chronic peritonitis, or post-peritoneal abscess. (3) Amœbic abscess may arise from direct or from portal infection. (4) Multiple liver abscesses are primarily bacillary. (5) Shiga's bacillus is the cause of ordinary dysentery, which is differentiated from the amœbic form by a "clumping" blood reaction.

[1] Brit. Med. Jour., April 18, 1903, p. 893. [2] Lancet, May 16, 1903, p. 1390. [3] Ibid., April 18, 1903, p. 1099. [4] Ibid., p. 1119. [5] Ibid., Jan. 24, 1903, p. 256. [6] Ibid., May 23, 1903, p. 1428. [7] Ibid., May 2, 1903, p. 1230. [8] Amer. Jour. Med. Sci., Feb., 1903, p. 215. [9] Lancet, May 2, 1903, p. 1222. [10] Brit. Med. Jour. Epitome, Feb. 14, 1903. [11] Brit. Med. Jour., June 6, 1903, p. 1315.

*(To be continued.)*

